# Affine Subspaces of Curvature Functions from Closed Planar Curves

**DOI:** 10.1007/s00025-021-01378-6

**Published:** 2021-03-20

**Authors:** Leonardo Alese

**Affiliations:** Department of Mathematics, TU Graz, Kopernikusgasse 24, 8010 Graz, Austria

**Keywords:** Closed curves, interpolation of curvature, Primary 53A04

## Abstract

Given a pair of real functions (*k*, *f*), we study the conditions they must satisfy for $k+\lambda f$ to be the curvature in the arc-length of a closed planar curve for all real $\lambda$. Several equivalent conditions are pointed out, certain periodic behaviours are shown as essential and a family of such pairs is explicitely constructed. The discrete counterpart of the problem is also studied.

## Introduction

Closed curves are natural mathematical objects which have been studied since a long time. In his well-known paper [3] Fenchel makes the following comment about the study of the geometric properties of a space curve which depend on the assumption that the curve is closed.

*The results are often comparatively elementary and seem to be isolated. On the other hand, the intuitive character of the statements and the lack of a general method of approach make the field attractive...*

In this paper we focus on closed planar curves but the consideration above identifies pretty well the context of our contribution. In this area interesting questions keep on coming up, as in the recent [1], where a surprising result on permuting arcs of a $C^1$ curve with the goal to make the curve closed is proven with elementary topological tools.

The natural and complete geometric descriptor we associate to a curve is its *curvature*. If $\gamma \in C^2([0,2\pi ],\mathbb {R}^2)$ is an *arc-length parametrized planar curve*, i.e., a twice differentiable function from the interval $I:=[0,2\pi ]$ to the real plane such that the norm of its first derivative $\Vert \gamma ' \Vert$ is constantly equal to 1, we can define a *turning angle* function $\theta$ that satisfies $\gamma '(t)=(\cos \theta (t), \sin \theta (t))=\mathrm {e}^{i\theta (t)}$, where $\mathbb {R}^2$ has been identified with the complex plane $\mathbb {C}$. The *curvature* *k* of $\gamma$ is defined as the first derivative $\theta '$ of the turning angle function. The other way round, given a continuous curvature $k\in C^0(I, \mathbb {R})$ we can reconstruct by integration, uniquely up to rigid motions, the curve it comes from. In fact, $\theta (t)=\int _0^t k(s)\mathrm {d}s + C$ and $\gamma (t)=\int _0^t \mathrm {e}^{i\theta (s)}\mathrm {d}s + V$. For a more extensive treatment of the subject the reader may refer to [2].

Given another function $f\in C^0(I,\mathbb {R})$, the main question we are interested in this paper is:

What are the conditions on *k* and *f* for $k+\lambda f$ to be the curvature of a closed curve for all $\lambda \in \mathbb {R}$?

**Fig. 1:**
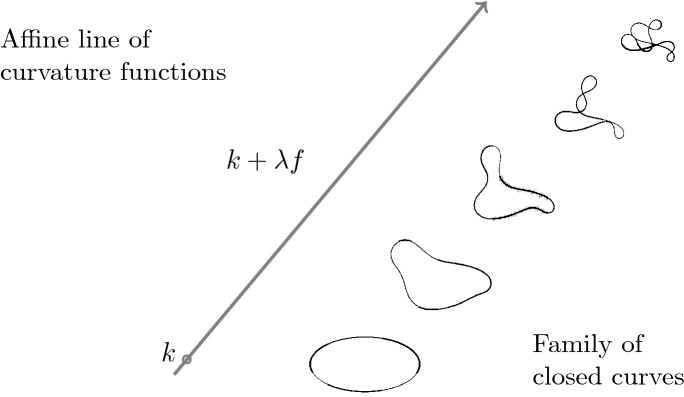
An ellipse is deformed by adding multiple of *f* to its curvature *k*. If, as in this case, *f* is chosen properly, then all the curves of the family are closed. We want to study the constraints *k* and *f* must satisfy to present such a behaviour

Here and in the following with *closed* we just mean that starting and end point of the curve coincide (we will see though that the nature of the problem entails much stiffer relations also on the derivatives at the extreme points of the curve). Figure 1 visualizes the objects we are going to study.

Interpolation of curvature functions is a tool used in computer graphics to gradually transform one curve into another, while mantaining the length of the curve [6]. This method does not perform well when it comes to deform closed curves, since there is no guarantee that the intermediate curves are closed as well; from the point of view of computer graphics this problem can be fixed by approximating the transition curves with closed ones that are not too far away from them [5]. In this paper we approach the problem from the theoretical perspective, exploring the conditions guaranteeing that all the curves are closed over the interpolation of the curvature functions.

As for an outline of the contents, Sect. 2 presents the main characterization theorem, proving also that the existence of a single affine line of curvature functions from closed curves is equivalent to the existence of an infinite dimensional affine space of such functions. In Sect. 3 periodicity properties of *k* and *f* are shown. On the existence side, Sect. 4 deals with the explicit construction of pairs of analytic function (*k*, *f*) that satisfy our constraints. In Sect. 5 we conclude by discussing the discrete case.

## Equivalent Characterizations of Closedness

Let $\gamma$ be a closed $C^2$ curve defined on the interval $I=[0,2\pi ]$, $\theta$ its associated turning angle function and $k=\theta '$ its curvature. For $f\in C^0(I, \mathbb {R})$, we want to answer the question: what are the conditions on *f* for $k+\lambda f$ to be the curvature of a closed curve for all $\lambda \in \mathbb {R}$? Calling $\phi (t):=\int _0^t f(s)\mathrm {d}s$, this is equivalent to

$$\begin{aligned} \int _0^{2\pi } \mathrm {e}^{i(\theta (t)+\lambda \phi (t))} \mathrm {d}t=0, \quad \forall \; \lambda \in \mathbb {R}. \end{aligned}$$

The function $F(\lambda ):=\int _0^{2\pi } \mathrm {e}^{i(\theta (t)+\lambda \phi (t))}\mathrm {d}t$ is analytic in $\lambda$. This can be seen for example by giving the following explicit entire expansion for the real part of *F* (the imaginary part is analogous):

$$\begin{aligned} F_1(\lambda )=\sum _{c=0}^{\infty } c_n \frac{\lambda ^n}{n!}, \;\; \text {with } c_n = {\left\{ \begin{array}{ll} (-1)^{\frac{n}{2}} \;\;\; \int _0^{2\pi } \phi (t)^n \cos \theta (t) \mathrm {d}t, &{} \;\; \text {if } n \text { is even}, \\ (-1)^{\frac{n+1}{2}} \int _0^{2\pi } \phi (t)^n \sin \theta (t)\mathrm {d}t, &{} \;\; \text {if } n \text { is odd}. \end{array}\right. } \end{aligned}$$

This observation alone is enough to conclude the first of our equivalent conditions.

### Lemma 2.1

Let $k,f \in C^0(I,\mathbb {R})$. Then the curve with curvature $k+\lambda f$ is closed $\forall \; \lambda \in \mathbb {R}$ $\Leftrightarrow$ we have the equality

1 $$\begin{aligned} \int _0^{2\pi } \mathrm {e} ^{i \theta (t)} \phi (t)^n \mathrm {d}t=0, \quad \forall \; n \in \mathbb {N}_0, \end{aligned}$$

where $$\theta (t)=\int _0^t k(s)\mathrm {d}s$$ and $$\phi (t) = \int _0^t f(s) \mathrm {d}s$$.

### Proof

An analytic function is everywhere 0 if and only if all of its derivatives vanish in at least one point. We conclude by computing the *n*-th derivative of *F* and evaluating it in $\lambda =0$, obtaining $0=F^{(n)}(0)= i^n \int _0^{2\pi } \mathrm {e} ^{i \theta (t)} \phi (t)^n \mathrm {d}t$. Note that we could take the derivative within the integral thanks to the Leibniz integral rule. $\square$

We want now to better understand this condition, by discussing some of its implications. Our main tool will be an approximation argument based on the observation that, if $\phi$ satisfies the condition above, then for any $N \in \mathbb {N}_0$ and $(c_j)_{j\in \{0,\ldots ,N\}} \in \mathbb {R}^{N+1}$, also the sum $\sum _{j=0}^N c_j \phi ^j$ does.

### Lemma 2.2

$\theta ,\phi \in C^1(I,\mathbb {R})$ satisfy condition (1) $\Leftrightarrow$ $\theta ,g(\phi )$ (composition of functions) satisfy condition (1), for any *g* bounded and integrable.

### Proof

The ‘if’ part is trivial. For the ‘only if’ we use a density property of polynomials in our class of functions to approximate *g*. More explicitely, for any $\varepsilon > 0$, there exists a polynomial $p_{\varepsilon }$ of degree $N(\varepsilon )$ such that

$$\begin{aligned} \int _0^{2\pi } \big | g(\phi (t)) - p_{\varepsilon }(\phi (t)) \big | \mathrm {d}t <\varepsilon , \end{aligned}$$

which implies

$$\begin{aligned}&\left|\int _0^{2\pi } \mathrm {e}^{i \theta (t)} g(\phi (t)) \mathrm {d}t \right|\\ \le&\left|\int _0^{2\pi } \mathrm {e}^{i \theta (t)} \bigl ( g (\phi (t)) - p_{\varepsilon }(\phi (t))\bigl ) \mathrm {d}t\right|+ \left| \int _0^{2\pi } \mathrm {e}^{i \theta (t)} p_{\varepsilon }(\phi (t)) \mathrm {d}t \right| \\ \le&\int _0^{2\pi } \big | g (\phi (t)) - p_{\varepsilon }(\phi (t)) \big | \mathrm {d}t \le \varepsilon . \end{aligned}$$

$$\square $$

By Lemma 2.2, the existence of $\phi$ satisfying (1) implies the existence of an infinite-dimensional affine space through $\theta$ whose elements satisfy (1) as well. From the perspective of the curvature, what we are saying here is that, choosing *g* to be $C^1$, we can pass from *f* to $fg'(\phi )$ and still have that the curves with curvature functions $k+\lambda fg'(\phi )$ are closed for all $\lambda$.

Before moving to the next lemma, which provides a much more local characterization of our constraint, it is convenient to recall that a level set $\phi ^{-1}(a)=\{t \mid \phi (t)=a \}$ consists of isolated points, if $\phi '(t)\ne 0$ for all $t \in \phi ^{-1}(a)$. For $\phi$ defined on a compact interval *I*, level sets of such regular values are therefore finite.

### Lemma 2.3

If $\theta ,\phi \in C^1(I,\mathbb {R})$ satisfy condition (1), then we have the implication

2 $$\begin{aligned} a \ne \phi (0),\phi (2\pi ) \text { is a regular value of } \phi \Rightarrow \sum _{b\in \phi ^{-1}(a)} \frac{\mathrm {e}^{i\theta (b)}}{|\phi '(b)|}=0. \end{aligned}$$

### Proof

In Lemma 2.2 we select $g=\chi _{[a,a+\delta ]}$, that is the characteristic function of the interval $[a,a+\delta ]$. We can find $\delta >0$ such that the restrictions $\{\phi _j\}$ of $\phi$ to the finitely many components $\{ [t_{1,j}(\delta ),t_{2,j}(\delta )] \}$ of $\phi ^{-1}([a,a+\delta ])$ are invertible and obtain

$$\begin{aligned} \sum _j \int _{t_{1,j}(\delta )}^{t_{2,j}(\delta )} \mathrm {e}^{i \theta (t)} \phi (t) \mathrm {d}t = 0. \end{aligned}$$

We then take the derivative with respect to $\delta$, which must be 0 as the function is constant, providing

$$\begin{aligned} \sum _j \mathrm {e}^{\theta (\phi _j^{-1}(a+\delta ))} \phi (\phi _j^{-1}(a+\delta )) \cdot |(\phi _j^{-1})'(a+\delta )|=0. \end{aligned}$$

We finally evaluate the result at $\delta =0$ concluding

$$\begin{aligned} a \sum _{b\in \phi ^{-1}(a)} \frac{\mathrm {e}^{i\theta (b)}}{|\phi '(b)|}=0. \end{aligned}$$

$$\square $$

The reason we excluded the level sets $\phi (0)$ and $\phi (2\pi )$ from the constraint on the sum is simply to avoid to distinguish cases depending on the sign of the derivative at extreme points of the interval.

### Remark 2.4

In order to have the equivalence (1) $\Leftrightarrow$ (2), in addition we must require $\int _{\phi ^{-1}(a)}\mathrm {e}^{i\theta }=0$, for all $a\in \mathbb {R}$. If $\phi$ is analytic this requirement is always met.

We collect in one theorem all the conditions we have proven equivalent.

### Theorem 2.5

Let $k,f \in C^0(I,\mathbb {R})$ and $\theta (t)=\int _0^t k(s)\mathrm {d}s, \phi (t) = \int _0^t f(s) \mathrm {d}s$. The following conditions are equivalent.

0. The curve with curvature $k+\lambda f$ is closed $\forall \; \lambda \in \mathbb {R}$,
1. $$\int _0^{2\pi } \mathrm {e} ^{i \theta (t)} \phi (t)^n \mathrm {d}t=0, \quad \forall \; n \in \mathbb {N}_0, $$
2. $$\int _0^{2\pi } \mathrm {e} ^{i \theta (t)} g( \phi (t) ) \mathrm {d}t=0, \quad \forall \; n\in \mathbb {N}_0 \text { and any } g \text { bounded and integrable}$$.

Moreover, they imply

$$\begin{aligned} a \ne \phi (0),\phi (2\pi ) \text { is a regular value of } \phi \Rightarrow \sum _{b\in \phi ^{-1}(a)} \frac{\mathrm {e}^{i\theta (b)}}{|\phi '(b)|}=0. \end{aligned}$$

We also point out the following corollary, which rules out the possibility of vector spaces of curvatures of closed curves.

### Corollary 1

For $f \in C^0(I,\mathbb {R})$, there exists $\lambda$ such that the curve that has $\lambda f$ as curvature is not closed. More precisely, the set $\Lambda =\{ \lambda \in \mathbb {R} : \lambda f$ is the curvature of a closed curve$\}$ does not have accumulation points.

### Proof

Setting $k\equiv 0$, condition (1) of Theorem 2.5 becomes $\int _0^{2\pi } \phi (t)^n dt=0$ for all $n\in \mathbb {N}_0$, which for *n* even can only be satisfied by $\phi \equiv 0$. On the other hand, the presence of accumulation points in $\Lambda$ is enough to guarantee $\Lambda =\mathbb {R}$ by the analiticity argument from the beginning of the section, hence entailing the same conclusion. $\square$

## Conditions on the Boundary

In this section we discuss some periodicity properties that $\theta$ and $\phi$ must satisfy if the curve with turning angle function $\theta +\lambda \phi$ is closed for all $\lambda \in \mathbb {R}$. We will show that, under the conditions of Theorem 2.5, the respective behaviour of $\theta$ and $\phi$ on the boundary is strongly related.

### Proposition 3.1

If $\theta ,\phi \in C^h(I,\mathbb {R})$ satisfy condition (2) and $\phi (0)$ is not a critical value, then $\phi (0)=\phi (2\pi )$ and the derivatives of $\phi ,\theta$ obey either

$$\begin{aligned} \theta (2\pi )-\theta (0)\equiv 0 \mod 2\pi , \;\;&\theta ^{(k)}(0)= \theta ^{(k)}(2\pi ), \;&\quad \quad 1\le k \le h-1, \\&\phi ^{(k)}(0)= \phi ^{(k)}(2\pi ),&\quad \quad 1\le k \le h \end{aligned}$$

or

$$\begin{aligned} \theta (2\pi )-\theta (0)\equiv \pi \mod 2\pi , \;\;&\theta ^{(k)}(0)= (-1)^k \theta ^{(k)}(2\pi ), \;&1\le k \le h-1, \\&\phi ^{(k)}(0)= (-1)^k \phi ^{(k)}(2\pi ), \;&1 \le k \le h. \end{aligned}$$

### Proof

**Fig. 2:**
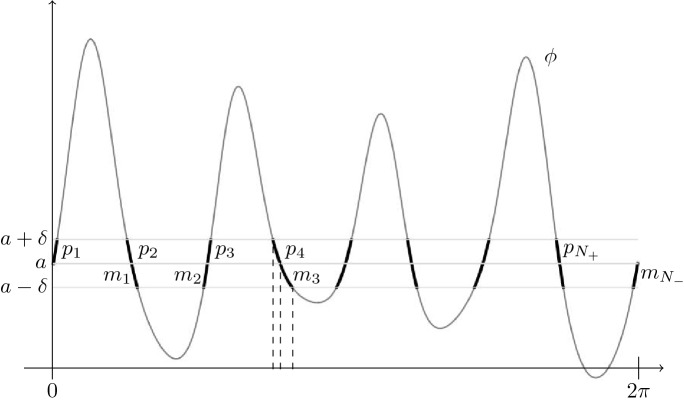
The functions $\{p_j\}_{j \in \{1,\ldots ,N_+\}}$ are the invertible restrictions of $\phi$ to the finitely many components of $\phi ^{-1}([a,a+\delta ])$ and analogously $\{m_j\}_{j \in \{1,\ldots ,N_-\}}$ are the restrictions of $\phi$ to $\phi ^{-1}([a-\delta ,a])$

Setting $a=\phi (0)$, we consider $\delta >0$ such that $\phi$ is invertible on the finitely many components of $\phi ^{-1}([a-\delta ,a+\delta ])$. We then look at the connected components of $\phi ^{-1}([a,a+\delta ])$ and we use the symbol $p_j$ for the restriction of $\phi$ to the *j*-th component, numbered from the left ($j=1,\ldots ,N_+$, see Figure 2). Similarly, functions $m_j$’s are the restriction of $\phi$ to the connected components of $\phi ([a-\delta ,a])$. Rewriting condition (2) we have

$$\begin{aligned} \lim _{\varepsilon \rightarrow 0^+} \sum _j \frac{\mathrm {e}^{i\theta \big (p_j^{-1}(a+\varepsilon )\big )}}{|\phi '(p_j^{-1}(a+\varepsilon ))|} = \lim _{\varepsilon \rightarrow 0^-} \sum _j \frac{\mathrm {e}^{i\theta \big (m_j^{-1}(a+\varepsilon )\big )}}{|\phi '(m_j^{-1}(a+\varepsilon ))|}=0. \end{aligned}$$

Since limit contributions coming from the restrictions to intervals in the interior of *I* are equal in the two sums, we have no other choice than $\phi (0)=\phi (2\pi )$, otherwise the contribution from $p_1$ in the first sum could not be balanced in the limit by any terms of the second sum. Without loss of generality we can assume $\phi '(0)>0$. We distinguish two cases, depending on the sign of $\phi '(2\pi )$. If $\phi '(2\pi )>0$, just by rewriting again condition (2) while keeping contributions from the two extreme intervals on the left-hand side of the equalities, we have , for $0< \varepsilon <\delta$

$$\begin{aligned} \frac{\mathrm {e}^{i\theta \big (p_1^{-1}(a+\varepsilon )\big )}}{\phi '(p_1^{-1}(a+\varepsilon ))}&=- \; \sum _{1<j} \frac{\mathrm {e}^{i\theta \big (p_j^{-1}(a+\varepsilon )\big )}}{|\phi '(p_j^{-1}(a+\varepsilon ))|}, \\ \frac{\mathrm {e}^{i\theta \big (m_{N_-}^{-1}(a-\varepsilon )\big )}}{\phi '(m_{N_-}^{-1}(a-\varepsilon ))}&=-\sum _{j<N_-} \frac{\mathrm {e}^{i\theta \big (m_j^{-1}(a-\varepsilon )\big )}}{|\phi '(m_j^{-1}(a-\varepsilon ))|}. \end{aligned}$$

The sums on the right-hand side of the equations are equal for $\varepsilon = 0$, entailing

$$\begin{aligned} \frac{\mathrm {e}^{i\theta (0)}}{\phi '(0)}=\frac{\mathrm {e}^{i\theta (2\pi )}}{\phi '(2\pi )}, \end{aligned}$$

which proves $\theta (2\pi )-\theta (0)\equiv 0 \mod 2\pi$ and $\phi '(0)=\phi '(2\pi )$. Analogously, taking the first derivative of the two equalities above with respect to $\varepsilon$ and considering the limit $\varepsilon \rightarrow 0$, we conclude

$$\begin{aligned} \frac{i\mathrm {e}^{i\theta (0)}\theta '(0)-\mathrm {e}^{i\theta (0)}\phi ''(0)\frac{1}{\phi '(0)}}{\phi '(0)^2}=\frac{i\mathrm {e}^{i\theta (2\pi )}\theta '(2\pi )-\mathrm {e}^{i\theta (2\pi )}\phi ''(2\pi )\frac{1}{\phi '(2\pi )}}{\phi '(2\pi )^2}, \end{aligned}$$

which, already knowing the respective relations of $\theta , \phi$ and $\phi '$ at extreme parameters, and noticing that $\mathrm {e}^{i\theta (0)}$ and $i \mathrm {e}^{i\theta (0)}$ are orthogonal, implies $\theta '(0)=\theta '(2\pi )$ and $\phi ''(0)=\phi ''(2\pi )$. For the derivatives of higher order, the statement follows analogously by induction.

If $\phi '(2\pi )<0$, we get

$$\begin{aligned} \frac{\mathrm {e}^{i\theta \big (p_1^{-1}(a+\varepsilon )\big )}}{\phi '(p_1^{-1}(a+\varepsilon ))} -\frac{\mathrm {e}^{i\theta \big (p_{N_+}^{-1}(a+\varepsilon )\big )}}{\phi '(p_{N_+}^{-1}(a+\varepsilon ))}&=-\sum _{1<j<N_+} \frac{\mathrm {e}^{i\theta \big (p_j^{-1}(a+\varepsilon )\big )}}{|\phi '(p_j^{-1}(a+\varepsilon ))|}, \\ 0&= - \;\;\; \sum _{j} \;\;\, \frac{\mathrm {e}^{i\theta \big (m_j^{-1}(a-\varepsilon )\big )}}{|\phi '(m_j^{-1}(a-\varepsilon ))|} \end{aligned}$$

and we conclude again by taking derivatives term by term with respect to $\varepsilon$ and using induction. $\square$

### Remark 3.2

Note that the constraint on the curves associated to $k+\lambda f$ to be closed for all $\lambda$ just means that starting and end point coincide. Proposition 3.1 proves that in this case the function *k* and *f* enjoy much stronger periodicity.

### Remark 3.3

In the hypotheses of Proposition 3.1, it holds $\phi (2\pi ) =\phi (0)$ but also, by definition of $\phi$, $\phi (0)=\int _0^{0} f(s) \mathrm {d}s = 0$. This means that along the affine line $k+\lambda f$ the total turning angle of the associated curve is constant and equal to 0 or $\pi$ up to multiples of $2\pi$.

## Explicit Constructions of Families of Closed Curves

In Sects. 2 and 3 we characterized pairs of functions (*k*, *f*) such that the curve obtained by integrating the curvature $k+\lambda f$ is closed for all $\lambda \in \mathbb {R}$. In this section we are interested in the existence of such pairs. We show how one can explicitly construct curvature functions with the desired properties.

### Lemma 4.1

If $$\theta \in C^1(I,\mathbb {R})$$, then

$$\begin{aligned} \exists \phi \in C^1(I,\mathbb {R}): \int _0^{2\pi }&\mathrm {e}^{i\theta (s)} \phi (s)^n \mathrm {d} s=0, \; \forall n\in \mathbb {N} \\&\Leftrightarrow \\ \exists \psi \in C^1(I,\mathbb {R}): \int _0^{2\pi }&\mathrm {e}^{i\theta (s)} \mathrm {e}^{i n \psi (s)} \mathrm {d}s=0, \; \forall n\in \mathbb {N}. \end{aligned}$$

### Proof

The first existence statement implies the second just by taking $\psi =\phi$ and recalling condition (2) of Theorem 2.5, guaranteeing that the composition with a function that is bounded and integrable mantains the desired property. The other way round we pick for example $\phi =\cos (\psi )$ and conclude by observing that $\cos (\psi )^n$ can be rewritten as a linear combination of terms of the form $\cos (h\cdot \psi )$. $\square$

We now consider curves allowing a periodic regular parametrization that can be expressed as a Fourier series with periodic gaps in the coefficients

$$\begin{aligned} \gamma (t)=\Bigg (\sum _{j=0}^\infty a_j \cos (j\cdot t)+b_j \sin (j\cdot t),\sum _{j=0}^\infty \bar{a}_j \cos (j\cdot t)+\bar{b}_j \sin (j\cdot t)\Bigg ), \end{aligned}$$

that is $a_j=b_j=\bar{a}_j=\bar{b}_j=0$ whenever *j* is an integer multiple of $M\in \mathbb {N}$. The asymptotics of the coefficients for *j* going to infinity determines periodicity and differentiability of the function (see for example [4]). From now on we assume that $\gamma$ is a closed analytic curve, which, in the most trivial case, can simply be obtained by truncating the series and considering a trigonometric polynomial; in this case all the harmonics with index larger than the degree of the polynomial are 0 and therefore there exists always *M* satisfying the conditions above. By the orthogonality relations between elements of a Fourier basis, writing $\gamma '(t)$ as $v(t)\mathrm {e}^{i\theta (t)}$ where $v(t)=\Vert \gamma '(t)\Vert$ is the speed of $\gamma$ and $\theta$ is the turning angle associated to the parametrization, we have

$$\begin{aligned} \int _0^{2\pi } v(t)\mathrm {e}^{i\theta (t)} \mathrm {e}^{i n\cdot M\cdot t} dt =0, \; \forall n \in \mathbb {N}. \end{aligned}$$

After reparametrizing with respect to the arc-length (always possible as long as the curve is regular) we obtain, possibly scaling the curve to a length of $2\pi$,

$$\begin{aligned} \int _0^{2\pi } \mathrm {e}^{i\theta (t(s))} \mathrm {e}^{i n\cdot M\cdot t(s)} ds =0, \; \forall n \in \mathbb {N}. \end{aligned}$$

**Fig. 3:**
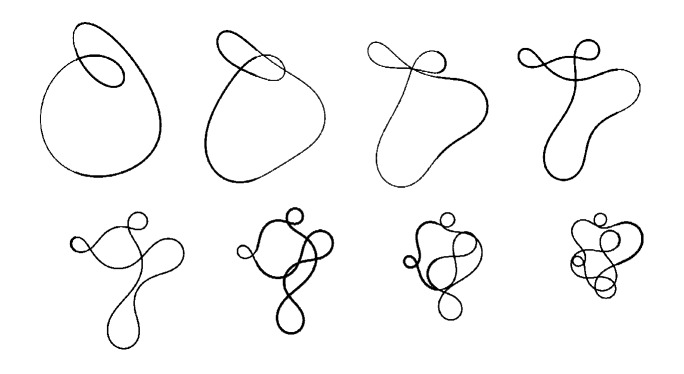
The turning angle $\theta$ of a trigonometric curve of degree 3 is linearly changed to $\theta +\lambda \phi$ with $\phi (t)=\mathrm {e}^{\cos (4t)}+2\cos (4t)$ while the curve remains closed. From top left to bottom right $\lambda$ goes from 0 to 0.7 by 0.1 increments

Note that if $\gamma$ is analytic than also $\gamma '$, $\Vert \gamma '\Vert$, $\int _0^t \Vert \gamma '\Vert$ and its inverse are analytic and therefore arc-length parametrization preserves analyticity. By the proof of Lemma 4.1, for example $\phi (s)=\cos (M \cdot t(s))$ and $\theta (t(s))$ satisfy condition (1) of Theorem 2.5 and therefore the analytic curve obtained by integrating $\mathrm {e}^{i(\theta +\lambda \phi )}$ is closed for all real $\lambda$’s (note that the functions $\phi$ and $\theta$ constructed this way are in general not periodic of any period smaller than $2\pi$). It is enough to take the derivative with respect to *s* to get the correspondent curvature functions. Figure 1 and Fig. 3 show families of curves obtained by such a linear modification of the turning angle (or equivalently of the curvature).

## The Discrete Case

In this section we look at a discretization of the problem we studied in the smooth setting. Consider an arc-length parametrized polyline, that is a finite sequence of *vertices* $(v_j)_{j \in \{1,2,\ldots ,N\}} \subset \mathbb {C}$ with $\Vert v_{j+1} - v_{j}\Vert =1$ for $1\le j \le N-1$. We define the *curvature* $k_j$ at a non-extreme vertex $v_j$ as the counter-clockwise angle between $v_{j} - v_{j-1}$ and $v_{j+1} - v_{j}$. The *turning angle* $\theta _j$ at an interior vertex $v_j$ is the sum $\sum _{r=2}^{j} k_r$. Also in this setting we can reconstruct, up to rigid motions, a polyline from its curvature, first computing the turning angle $(\theta _j)$ and then defining

$$\begin{aligned} v_1=0, \;\; v_2=1, \;\; v_j=v_{j-1}+\mathrm {e}^{i\theta _{j-1}} \text { for } j\ge 3. \end{aligned}$$

We consider now a polyline with *N* vertices, which is *closed* ($v_1=v_N$) and whose curvature is $(k_j)$. Given a discrete function $(f_j)_{j \in \{2,\ldots ,N-1\}} \in \mathbb {R}^{N-2}$, we ask what are the conditions on $(f_j)$ to guarantee that the polyline with curvature $(k_j)+\lambda (f_j)$ is closed for all $\lambda \in \mathbb {R}$. The following theorem answers this question, drawing a strong analogy to Theorem 2.5.

### Theorem 5.1

Let $(k_j)$ and $(f_j)$ be two discrete functions and $(\theta _j),(\phi _j)$ the turning angles obtained as their respective partial sums. The following conditions are equivalent

0. The polyline with curvature $(k_j)+\lambda (f_j)$ is closed $\forall \lambda \in \mathbb {R}$,
1. $$\sum _{1< j < N} \mathrm {e}^{i\theta _j} \phi _j^n = 0, \;\; \forall n \in \mathbb {N}_0$$,
2. $$\sum _{j \in \phi ^{-1}(a)} \mathrm {e}^{i\theta _j}=0, \;\; \forall a \in \mathbb {R}$$.

### Proof

The equivalence (0) $\Leftrightarrow$ (1) is deduced as in Sect. 2 by taking the *n*-th derivative with respect to $\lambda$ of the constant function $1=-\sum _{1< j < N} \mathrm {e}^{i(\theta _j+\lambda \phi _j)}$. Condition (1) is easily implied by (2), while for the opposite direction we observe that for all $n\in \mathbb {N}, a\in \mathbb {R} \setminus \{0\}$,

$$\begin{aligned} \sum _{1< j< N} \mathrm {e}^{i\theta _j} \bigg (\frac{\phi _j}{a}\bigg )^n=\frac{1}{a^n} \sum _{1< j < N} \mathrm {e}^{i\theta _j} \phi _j^n = 0. \end{aligned}$$

If all $\phi _j$’s are equal to 0, we are done since the polyline associated to the turning angles $\theta _j$ is closed. Otherwise, letting $A=\max _j \{ \left|\phi _j\right| \}$,

$$\begin{aligned} \begin{aligned} 0&=\lim _{n \rightarrow \infty } \sum _{1< j< N} \mathrm {e}^{i\theta _j} \bigg (\frac{\phi _j}{A}\bigg )^{2n} = \sum _{j \in \phi ^{-1}(A)} \mathrm {e}^{i\theta _j}+\sum _{j \in \phi ^{-1}(-A)} \mathrm {e}^{i\theta _j}, \\ 0&=\lim _{n \rightarrow \infty } \sum _{1< j < N} \mathrm {e}^{i\theta _j} \bigg (\frac{\phi _j}{A}\bigg )^{2n+1} = \sum _{j \in \phi ^{-1}(A)} \mathrm {e}^{i\theta _j}-\sum _{j \in \phi ^{-1}(-A)} \mathrm {e}^{i\theta _j}, \end{aligned} \end{aligned}$$

which entails $\sum _{j \in \phi ^{-1}(A)} \mathrm {e}^{i\theta _j}=\sum _{j \in \phi ^{-1}(-A)} \mathrm {e}^{i\theta _j}=0$. For $A'=\max _j \{ \left|\phi _j\right| \mid \left|\phi _j\right|<A \}$, it holds analogously

$$\begin{aligned} \begin{aligned} 0&=\lim _{n \rightarrow \infty } \sum _{1< j < N} \mathrm {e}^{i\theta _j} \bigg (\frac{\phi _j}{A'}\bigg )^n \\&= \lim _{n \rightarrow \infty } \sum _{j \not \in \phi ^{-1}(\pm A)} \mathrm {e}^{i\theta _j} \bigg (\frac{\phi _j}{A'}\bigg )^n+ \bigg (\frac{\pm A}{A'}\bigg )^n \sum _{j \in \phi ^{-1}(\pm A)} \mathrm {e}^{i\theta _j} \\&= \sum _{j \in \phi ^{-1}(A')} \mathrm {e}^{i\theta _j} \pm \sum _{j \in \phi ^{-1}(-A')} \mathrm {e}^{i\theta _j} +0, \end{aligned} \end{aligned}$$

and we conclude by iterating the same argument until we exhaust all the finitely many vertices of the polyline. $\square$

**Fig. 4:**
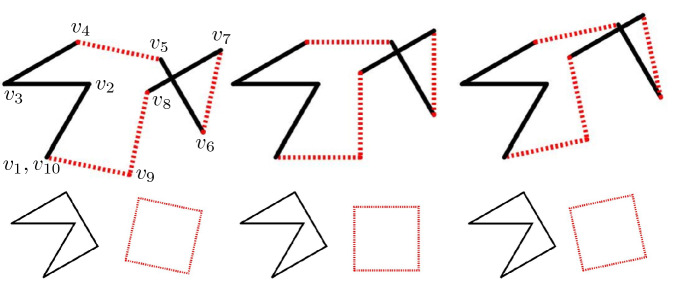
The discrete curvature $(k_j)$ of a polyline is modified linearly in $\lambda$ to $(k_j)+\lambda (f_j)$, with $(f_j)= (0,0,\phi _1,-\phi _1,\phi _1,$ $-\phi _1, \phi _1,0)$. Such a curvature vector sums up to the turning angle $(0,0,\phi _1,0,\phi _1,0,\phi _1,\phi _1)$, which rotates, as $\lambda$ varies, only the dashed edges corresponding to a balanced subset of indices

In order to find non-trivial pairs such that the polyline associated to $(k_j)+\lambda (f_j)$ is closed for all $\lambda$, by Theorem 5.1 the polyline associated to $(k_j)$ must possess at least one proper subset $\bar{V} \subset \{2,\ldots ,N-1\}$ of indices that is *balanced*, meaning $\sum _{j \in \bar{V}} \mathrm {e}^{i\theta _j}=0$. A visualization of this behaviour is given in Fig. 4.

Note that it is easy to construct polylines with no balanced proper subsets of edges. Consider for example *n* copies of the pair of unit vectors summing up to $(\frac{1}{n},0)$ and either the vector $(-1,0)$ for a polyline with an odd number of edges or the two unit vectors whose sum is $(-1,0)$ for an even number. Any proper subset of vectors from the “copies” part either consists of a single vector or its elements sum up to a non-unit vector different from 0. In both case it is not possible to counterbalance the sum with the vector(s) on the other side of the y-axis.

### Future Work

Given the curvature function *k* of a closed curve, when is it possible to find *f* such that the curve associated to $k+\lambda f$ is closed for all $\lambda$? In Sect. 4 we identified a class of pairs of functions that satisfies this condition and the next obvious step would be a full characterization in the $C^h$ and analytic setting. Thinking in terms of the turning angle $\theta =\int k$, a possible way of approaching the problem could be by synthesizing a Fourier series for $\phi$ that would satisfy the family of orthogonality relations $\int \mathrm {e}^{i\theta }\phi ^n=0$ in $L^2$. The ugliness of the convolution formula for the Fourier coefficients of a product prevented the author from succeeding.
